# A Global View of Gene Expression in the Aging Kidney

**DOI:** 10.1371/journal.pbio.0020451

**Published:** 2004-11-30

Four years ago in *Science*, Stuart Kim, a Stanford developmental biologist, made the case for laying down the broad strokes of a complex physiological process before defining its mechanisms. “A powerful, top-down, holistic approach,” he wrote, “is to identify all of the components of a particular cellular process, so that one can define the global picture first and then use it as a framework to understand how the individual components of the process fit together.” To get a broad view of gene expression in the aging nematode, Kim's lab turned to DNA microarrays and functional genomics. In a new study, Kim and colleagues apply this same approach to the decidedly more complex problem of human aging and “present a molecular portrait” of the aging kidney.

Scientists have identified a wide range of molecular pathways and mechanisms associated with aging. Many have been found in evolutionarily distant organisms, suggesting they have been conserved and could shed light on human aging. Yet other studies suggest that since few animals reach old age in the wild, any aging-related physiological changes aren't likely to impact the fitness of a population and so aren't likely to be conserved. Consequently, aging pathways in worms, for example, would have little bearing on humans. To investigate the molecular pathways associated with human aging, the authors focused on human tissue—in this case, the kidney.

Kidneys came from 74 patients, ranging in age from 27 to 92. Samples were extracted from donated kidneys or “meticulously harvested” from kidneys with localized disease to ensure only normal tissue was taken. Two structures that are critical to kidney function (removing toxins from blood) were removed from each sample: the renal cortex, which filters plasma, and the medulla, which alters urine composition to maintain fluid balance. Both deteriorate with age. An extensive clinical history was noted for each sample to account for any potentially confounding medical factors.[Fig pbio-0020451-g001]

**Figure pbio-0020451-g001:**
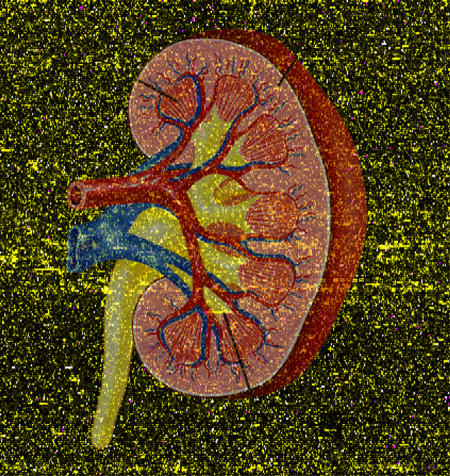
Transcriptional profiling to study aging in the kidney

Kim and colleagues then isolated RNA transcripts from the samples to determine the activity of every gene, broken down by age and kidney section, through microarray analysis. Looking for differences in gene expression across the genome, they identified genes that showed a statistically significant change in expression as a function of age. Of 33,000 known human genes on the microarray, 985 showed age-related changes, most showing increased activity. These changes are truly age-regulated, the authors conclude, since none of the medical factors impacted the observed changes in gene expression.

Although cortex and medulla have different cell types and perform different functions, their genetic aging profile was very similar, suggesting a common aging mechanism operates in both structures. In fact, these mechanisms may function broadly, as most of the age-regulated kidney genes were also active in a wide range of human tissues. Other organisms appear to lack these changes, however, prompting the authors to argue that understanding aging in humans will require human subjects.

Most importantly, the genetic profile of the tissue samples correlated with the physiological and morphological decline of an aging kidney. An 81-year-old patient with an unusually healthy kidney had a molecular profile typical of someone much younger, while a 78-year-old with a damaged kidney had the profile of a much older person. Using the power of functional genomics, this study has identified a set of genes that can serve as molecular markers for various stages of a deteriorating kidney and predict the relative health of a patient compared to their age group. These gene sets can also serve as probes to shed light on the molecular pathways at work in the aging kidney, and possibly on the process of aging itself.

